# Supplementary material for: Seasonal variations and co-occurrence networks of bacterial communities in the water and sediment of artificial habitat in Laoshan Bay, China
Source: PeerJ. 2022 Jan 4;10:e12705. doi: 10.7717/peerj.12705 (PMC8740510; doi:10.7717/peerj.12705)
Supplement: Supplemental Information 1 [file peerj-10-12705-s001.pdf]

Supplementary Files

Supp. File 1 The detailed procedures of the high-throughput sequencing and bioinformatics analyses

DNA extraction, PCR amplification and Illumina MiSeq sequencing

Genomic DNA was extracted using the FastDNA® SPIN Kit for Soil (MP Biomedicals, Irvine, CA, USA) according to the manufacturer's instructions. The DNA extract was checked on a 1% agarose gel, and the DNA concentration and purity were determined using a NanoDrop 2000 UV-vis spectrophotometer (Thermo Scientific, Wilmington, DE, USA). The extracted DNA samples were used for PCR amplification using the universal primers 338F (5'-ACTCCTACGGGAGGCAGCAG-3') and 806R (5'-GGACTACHVGGGTWTCTAAT-3'), which amplified the 468 bp V3-V4 hypervariable region of the 16S rRNA gene.

PCR amplification was performed as follows: initial denaturation at 95°C for 3 min, followed by 27 cycles of denaturing at 95°C for 30 s, annealing at 55°C for 30 s and extension at 72°C for 45 s, a single extension at 72°C for 10 min, and a final extension at 4°C. The PCR mix consisted of a 20 μL mixture containing 4 μL of 5 × *TransStart* FastPfu buffer, 2 μL of 2.5 mM dNTPs, 0.8 μL of forward primer (5 μM), 0.8 μL of reverse primer (5 μM), 0.4 μL of *TransStart* FastPfu DNA Polymerase, 10 ng of template DNA, and a ddH_2O volume up to 20 μL. The PCRs were performed in triplicate. The PCR product was run on a 2% agarose gel, and the amplicons were then extracted and purified using the AxyPrep DNA Gel Extraction Kit (Axygen Biosciences, Union City, CA, USA) according to the manufacturer's instructions. Subsequently, the DNA was quantified using the Quantus™ Fluorometer (Promega, Madison, WI, USA). Purified amplicons were pooled in equimolar and paired-end sequenced on an Illumina MiSeq PE300 platform (Illumina, San Diego,USA) according to the standard protocols by Majorbio Bio-Pharm Technology Co. Ltd. (Shanghai, China).

Sequence analysis

Raw sequence reads were analyzed using the QIIME 1.9 bioinformatics pipeline. Sequences shorter than 200 bp, quality scores lower than 20, and mismatched bases in barcodes or primers were removed. Operational taxonomic units (OTUs) were clustered with a 97% similarity cutoff using UPARSE 7.0, and chimeric sequences were identified and removed using UCHIME. The taxonomy of each sequence was analyzed by RDP Classifier 11.5 against the Silva database (v138). For downstream analyses, a randomly selected subset of the lowest sequencing number per sample was used to compare the relative differences among samples.

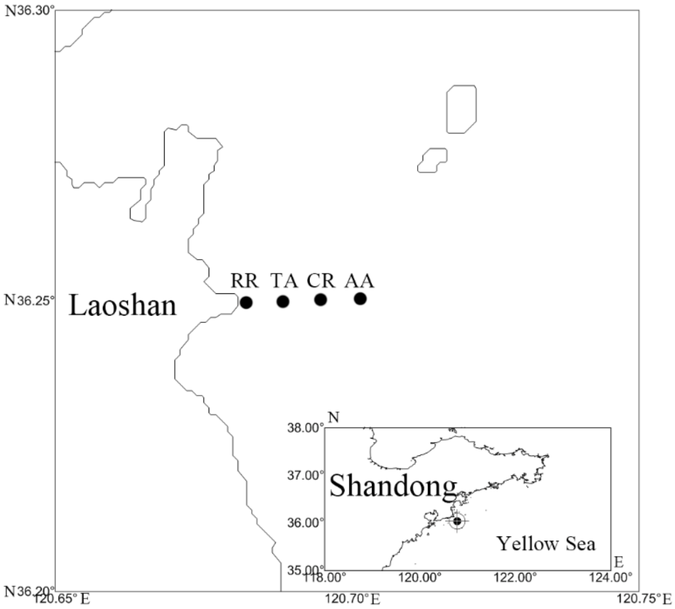

Fig. S1 Sampling areas in the Laoshan Bay artificial reefs (ARs).

Four sampling areas in ARs were studied: rock reefs (RR), transition areas (TA), concrete reefs (CR) and adjacent areas (AA).

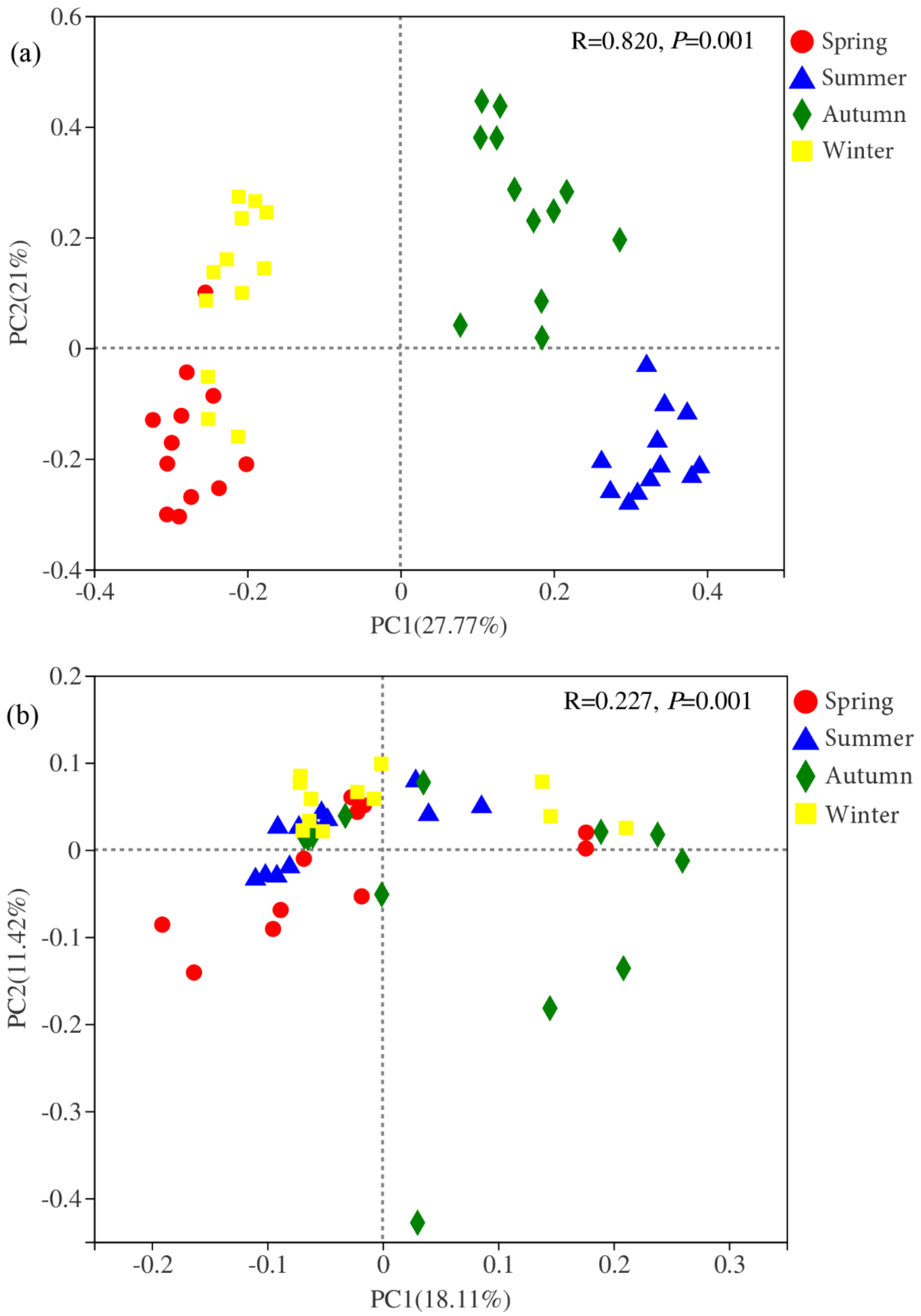

Fig. S2 Principal coordinates analysis (PCoA) plots of bacterial communities in the (a) water and (b) sediment of artificial reefs.

R: the test statistical significance of analysis of similarities (ANOSIM); *P*: statistical significance value at $\alpha = 0.05$ level.

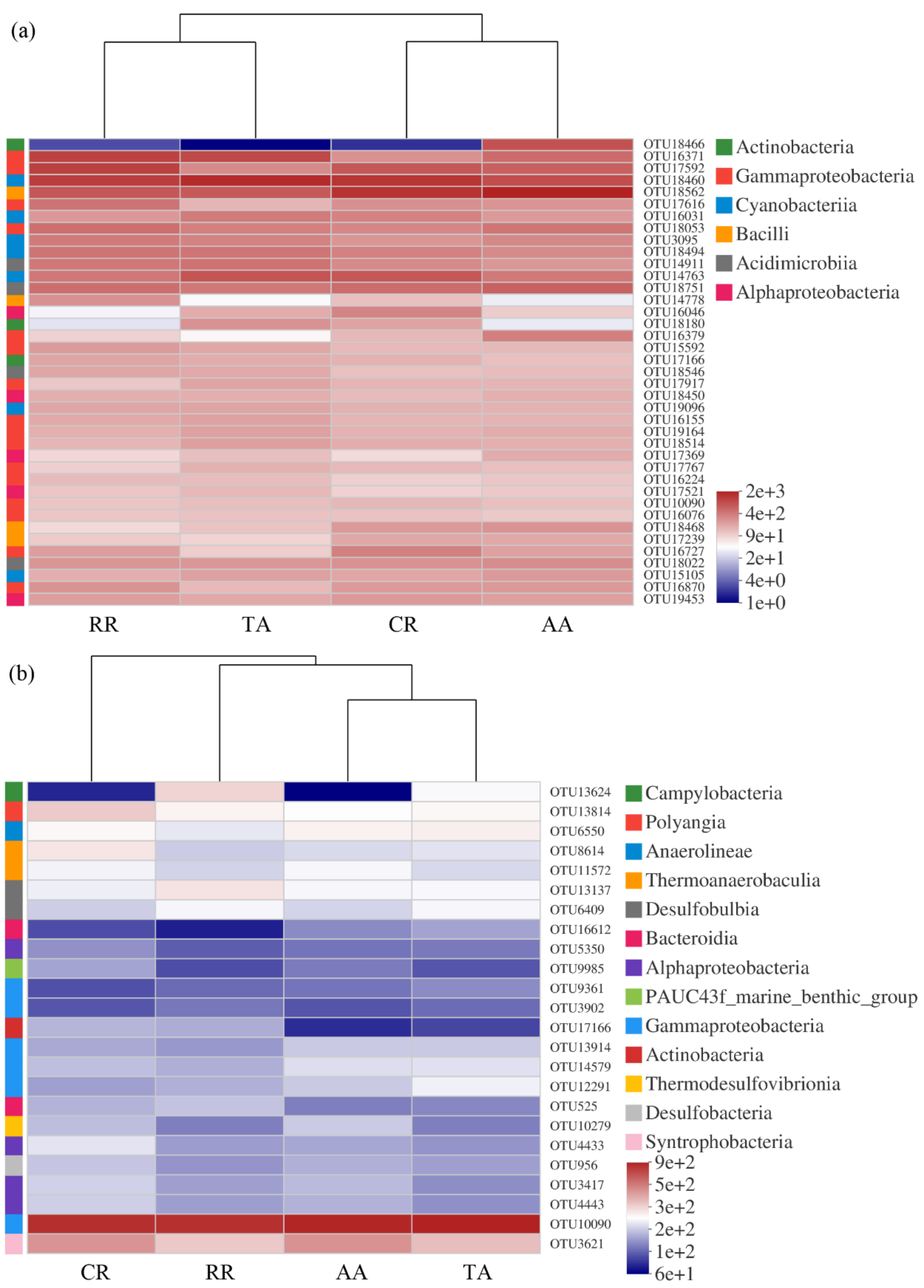

Fig. S3 Heatmap of top abundant OTUs of bacterial communities with sampling areas in the (a) water and (b) sediment of artificial reefs.

The cluster trees were analyzed to show the similarity of OTUs using Bray-Curtis distance. Four sampling areas in ARs were studied: rock reefs (RR), transition areas (TA), concrete reefs (CR) and adjacent areas (AA).

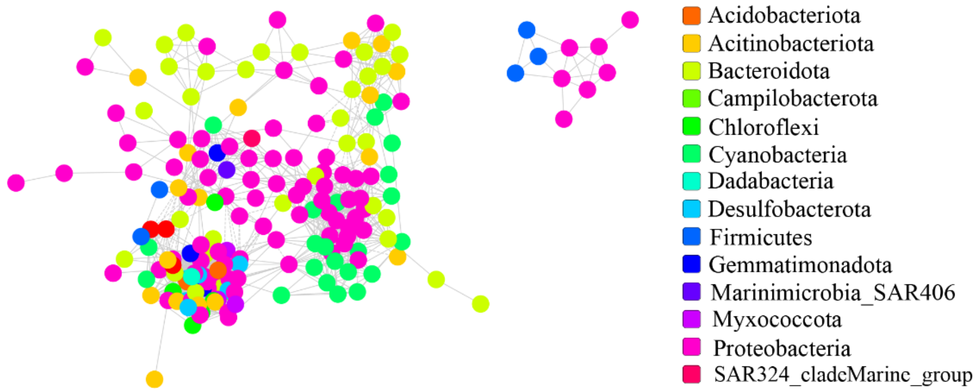

Fig. S4 Co-occurrence networks built from abundant bacterial OTUs in the water of artificial reefs.

Nodes are colored at phylum levels. Edges with $|r| \geqslant 0.8$ and $P \leqslant 0.001$ are shown in the networks. Positive and negative lines are represented by solid lines and dotted lines, respectively.

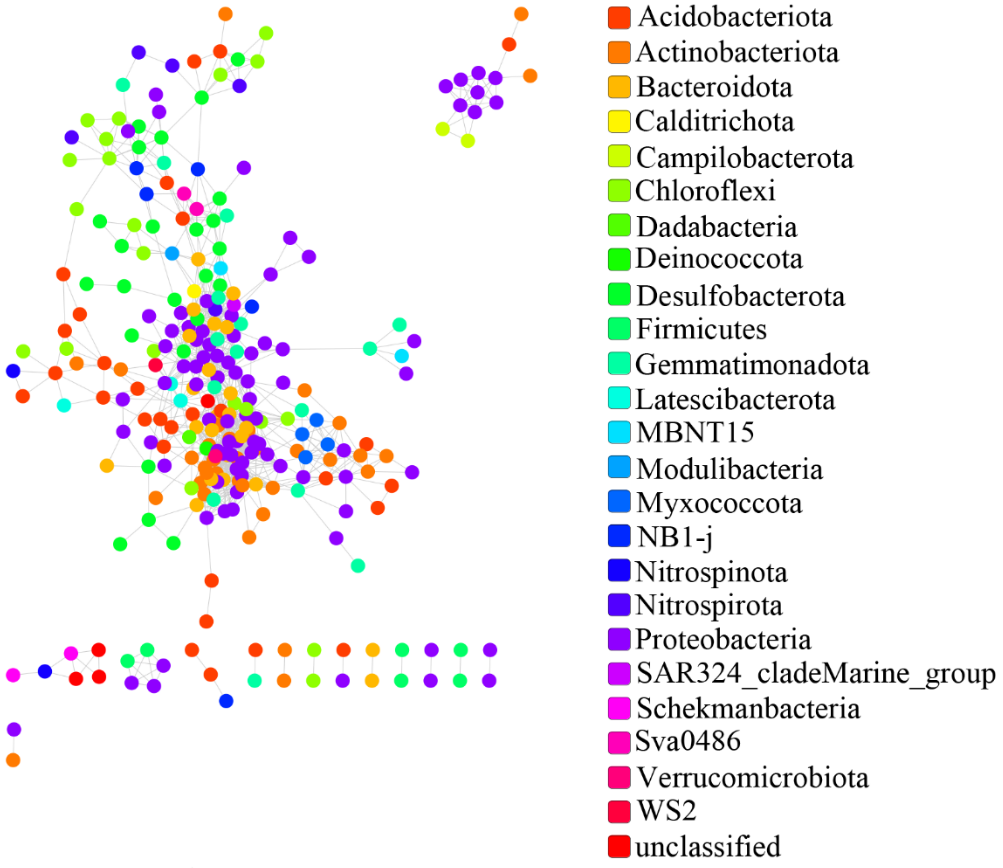

Fig. S5 Co-occurrence networks built from abundant bacterial OTUs in the sediment of artificial reefs.

Nodes are colored at phylum levels. Edges with $|r| \geqslant 0.8$ and $P \leqslant 0.001$ are shown in the networks. Positive and negative lines are represented by solid lines and dotted lines, respectively.

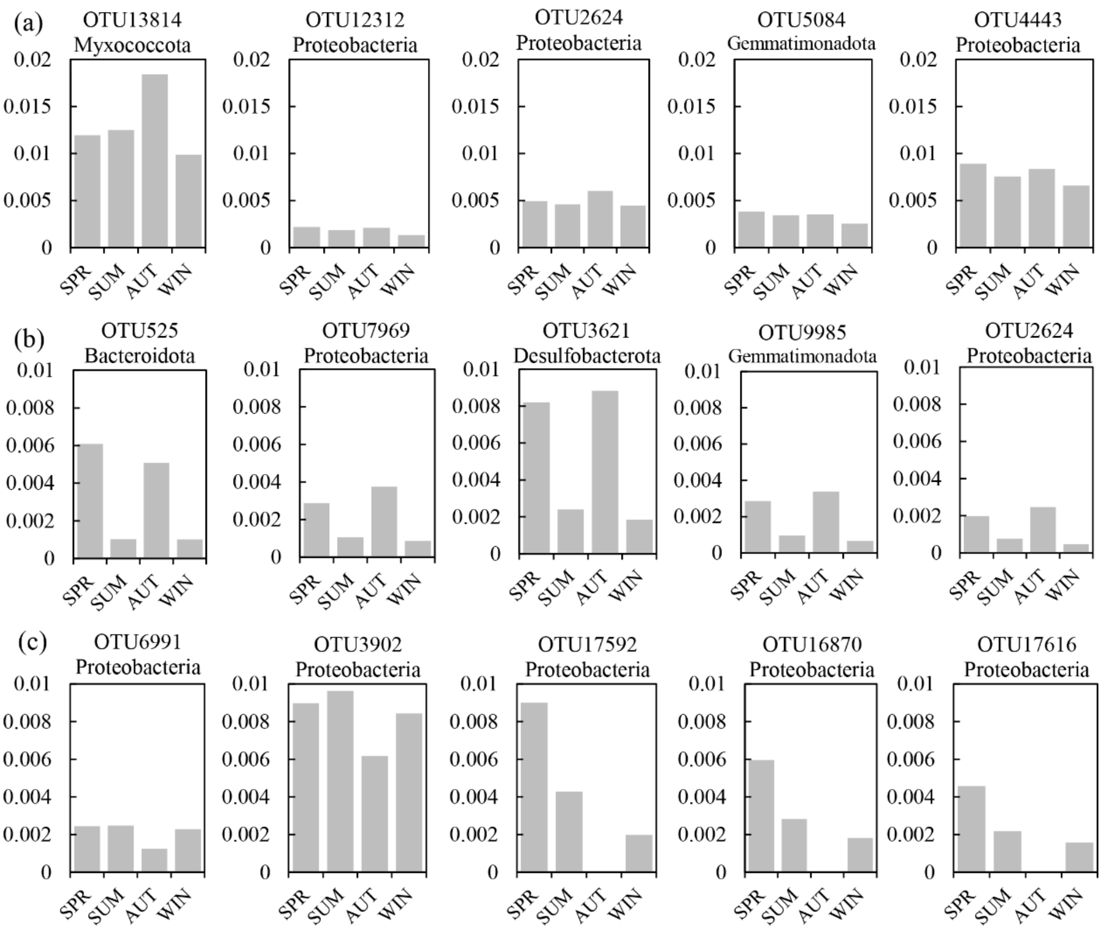

Fig. S6 Seasonal changes of the relative abundance for the top 5 keystone OTUs of the co-occurrence networks in the (a) water and sediment, (b) water and (c) sediment of artificial reefs.

Four seasons: spring (SPR); summer (SUM); autumn (AUT); winter (WIN).

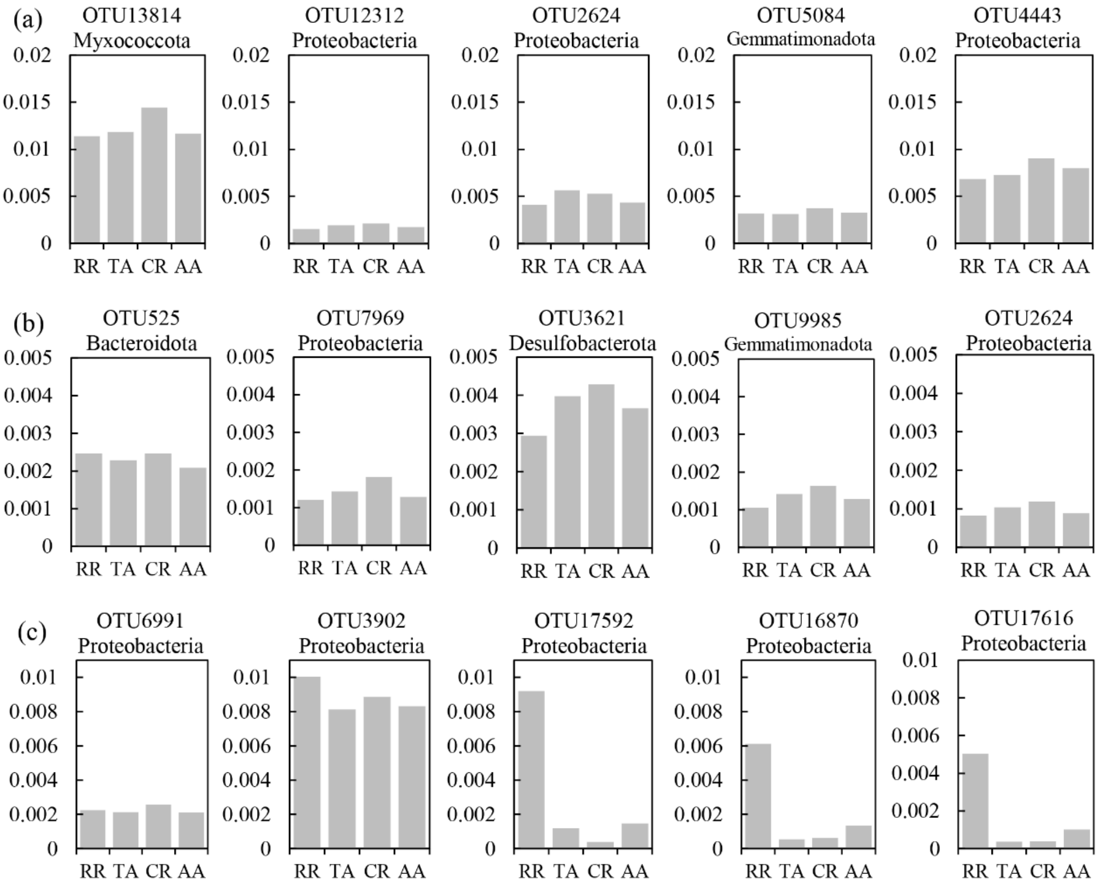

Fig. S7 Variations of the relative abundance for the top 5 keystone OTUs among four sampling areas of the co-occurrence networks in the (a) water and sediment, (b) water and (c) sediment of artificial reefs (ARs).

Four sampling areas in ARs: rock reefs (RR), transition areas (TA), concrete reefs (CR) and adjacent areas (AA).

Table S1a Environmental factors for the water of artificial reefs.

Environmental factors: chemical oxygen demand (COD); depth (Dep); dissolved oxygen (DO); transparency (Trans); chlorophyll-a (Chla); turbidity (Turb); active silicate (SiO_3); active phosphate (PO_4); nitrite (NO_2); nitrate (NO_3); ammonium (NH_4); suspended particulate materials (SPM); particulate organic matter (POM); total organic carbon (TOC); temperature (Temp); salinity (Sal).

Season	COD mg/L	Dep m	DO mg/L	Trans m	Chla ug/L	Turb NTU	SiO_3 mg/L	PO_4 mg/L	NO_2 mg/L	NO_3 mg/L	NH_4 mg/L	SPM ug/L	POM ug/L	TOC mg/L	Temp ℃	Sal	pH
Spring	0.629	9.667	8.452	2.317	0.789	3.987	0.031	0.011	1.794	0.389	0.036	39.780	7.46	2.726	14.625	32.11	7.97
Summer	0.525	10.151	8.649	1.092	0.885	4.823	0.079	0.030	1.628	0.188	0.141	32.574	6.59	2.662	24.942	31.42	8.06
Autumn	1.051	9.783	7.417	0.800	0.573	4.888	0.087	0.029	0.951	0.399	0.028	29.877	5.65	2.594	17.092	32.25	8.08
Winter	0.668	9.808	10.071	0.917	0.671	6.441	0.066	0.031	2.110	0.837	0.069	22.178	3.85	1.369	6.867	32.96	8.25

Table S1b Environmental factors for the sediment of artificial reefs.

Season	Mean particle um	Bulk density g/cm^3	Water content %	pH	Electrical conductivity mS/cm	Salinity	Organic matter content %	Mud content %
Spring	0.65	0.78	43.27	8.69	7.27	4.01	1.22	37.92
Summer	2.23	1.48	44.14	8.30	5.33	2.81	4.82	65.57
Autumn	2.25	1.55	41.83	8.47	7.32	4.09	5.51	68.96
Winter	0.90	1.50	49.01	8.38	4.63	8.26	1.93	78.25

Table S2a Top 10 keystone OTUs of bacterial co-occurrence network in the water and sediment.

Superscript in the Abundance represented the rank of relative abundance in the whole bacterial community.

OTU	Keystoneness	Phylum	Class	Order	Family	Abundance (‰)
OTU13814	0.840	Myxococcota	Polyangia	Polyangiales	Sandaracinaceae	12.3^{15}
OTU12312	0.836	Proteobacteria	Gammaproteobacteria	norank	norank	1.8^{150}
OTU2624	0.835	Proteobacteria	Gammaproteobacteria	B2M28	norank	4.8^{57}
OTU5084	0.835	Gemmatimonadota	PAUC43f_marine_benthic	norank	norank	3.3^{89}
OTU4443	0.835	Proteobacteria	Alphaproteobacteria	Kiloniellales	Kiloniellaceae	7.8^{29}
OTU1904	0.835	Myxococcota	Polyangia	Polyangiales	Sandaracinaceae	4.5^{63}
OTU17311	0.834	Chloroflexi	Anaerolineae	Caldilineales	Caldilineaceae	4.4^{64}
OTU5899	0.834	Actinobacteriota	Acidimicrobiia	Actinomarinales	norank	2.0^{133}
OTU8539	0.833	Actinobacteriota	Acidimicrobiia	Actinomarinales	norank	1.9^{147}
OTU10190	0.832	Proteobacteria	Gammaproteobacteria	unclassified	unclassified	1.9^{140}

Table S2b Top 10 keystone OTUs of bacterial co-occurrence network in the water.

Superscript in the Abundance represented the rank of relative abundance in the whole bacterial community.

OTU	Keystoneness	Phylum	Class	Order	Family	Abundance (‰)
OTU525	0.712	Bacteroidota	Bacteroidia	Flavobacteriales	Flavobacteriaceae	2.3^{90}
OTU7969	0.709	Proteobacteria	Gammaproteobacteria	Steroidobacterales	Woeseiaceae	1.4^{123}
OTU3621	0.708	Desulfobacterota	Syntrophobacteria	Syntrophobacterales	norank	3.7^{63}
OTU9985	0.708	Gemmatimonadota	PAUC43f_marine_benthic	norank	norank	1.3^{129}
OTU2624	0.708	Proteobacteria	Gammaproteobacteria	B2M28	norank	1.0^{149}
OTU8614	0.708	Acidobacteriota	Thermoanaerobaculia	Thermoanaerobaculales	Thermoanaerobaculaceae	3.1^{76}
OTU13137	0.702	Desulfobacterota	Desulfobulbia	Desulfobulbales	Desulfobulbaceae	1.4^{128}
OTU11572	0.702	Acidobacteriota	Thermoanaerobaculia	Thermoanaerobaculales	Thermoanaerobaculaceae	1.6^{119}
OTU16662	0.701	Bacteroidota	Bacteroidia	Cytophagales	Cyclobacteriaceae	1.9^{107}
OTU7413	0.700	Proteobacteria	Alphaproteobacteria	Rhodobacterales	Rhodobacteraceae	1.1^{137}

Table S2c Top 10 keystone OTUs of bacterial co-occurrence networks in the sediment.

Superscript in the Abundance represented the rank of relative abundance in the whole bacterial community.

OTU	Keystoneness	Phylum	Class	Order	Family	Abundance (‰)
OTU6991	0.722	Proteobacteria	Gammaproteobacteria	pItb-vmat-80	norank	2.3^{114}
OTU3902	0.704	Proteobacteria	Gammaproteobacteria	Steroidobacterales	Woeseiaceae	8.8^{24}
OTU17592	0.693	Proteobacteria	Gammaproteobacteria	Pseudomonadales	Moraxellaceae	3.1^{84}
OTU16870	0.693	Proteobacteria	Gammaproteobacteria	Pseudomonadales	Moraxellaceae	2.2^{121}
OTU17616	0.693	Proteobacteria	Gammaproteobacteria	Pseudomonadales	Moraxellaceae	1.7^{154}
OTU18562	0.693	Firmicutes	Bacilli	Bacillales	Planococcaceae	6.7^{34}
OTU18468	0.693	Firmicutes	Bacilli	Bacillales	Planococcaceae	1.6^{172}
OTU9719	0.671	Proteobacteria	Gammaproteobacteria	Gammaproteobacteria_Incertae_Sedis	unclassified	1.8^{151}
OTU12291	0.667	Proteobacteria	Gammaproteobacteria	Steroidobacterales	Woeseiaceae	14.6^{10}
OTU6550	0.667	Chloroflexi	Anaerolineae	Anaerolineales	Anaerolineaceae	19.0^{5}
